# Tryps and trips: cell trafficking across the 100-year-old blood–brain barrier

**DOI:** 10.1016/j.tins.2014.03.007

**Published:** 2014-06

**Authors:** Marina Bentivoglio, Krister Kristensson

**Affiliations:** 1 Department of Neurological and Movement Sciences, University of Verona, Verona, Italy; 2 Department of Neuroscience, Karolinska Institutet, Stockholm, Sweden

**Keywords:** neuroinflammation, neurovascular unit, endocytosis, pericytes, neurodegeneration, sleeping sickness

## Abstract

One hundred years ago, Edwin E. Goldmann discovered the blood–brain barrier (BBB) using trypan dyes. These dyes were developed and named by Paul Ehrlich during his search for drugs to kill African trypanosomes (extracellular parasites that cause sleeping sickness) while sparing host cells. For Ehrlich, this was the first strategy based on the ‘chemotherapy’ concept he had introduced. The discovery of the BBB revealed, however, the difficulties in drug delivery to the brain. Mechanisms by which parasites enter, dwell, and exit the brain currently provide novel views on cell trafficking across the BBB. These mechanisms also highlight the role of pericytes and endocytosis regulation in BBB functioning and in disrupted BBB gating, which may be involved in the pathogenesis of neurodegeneration.

## Highlights

• The blood–brain barrier (BBB) was discovered one century ago by the use of trypan dyes.
• The discovery initiated the targeted brain delivery of drugs.
• Trypan dyes were developed to kill African trypanosomes that cause sleeping sickness.
• Trypanosomes disclose cell trafficking in and out of the BBB.
• Disturbed gating at the BBB may cause neurodegeneration.

## Trypanosome attacks unveiled the BBB

**Figure 1:**
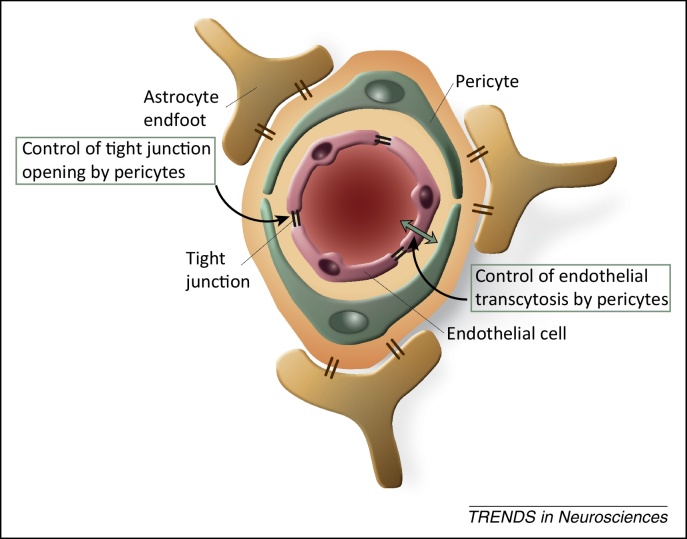
Schematic representation of the blood–brain barrier (BBB) and roles exerted by pericytes. At the interface between the systemic circulation and the central nervous system (CNS), the BBB is composed of highly specialized and polarized endothelial cells with tight junctions sealing the intercellular clefts, basement membranes, pericytes, astrocyte endfeet, with anchoring transmembrane proteins, which establish communication with neurons in the neurovascular unit. Astrocyte endfeet actually envelop almost the entire abluminal surface of CNS microvessels. Novel data indicate that pericytes, embedded within the endothelial cell-derived basement membrane, are involved in the control of endothelial transcytosis and tight junction opening. Adapted from [65].

As a drawbridge, the BBB protects the gatehouse of the central nervous system (CNS) castle, surrounded by the moat of the bloodstream. The castle loopholes are very tight, represented by tight junctions of cerebral microvessels (capillaries and postcapillary venules), assisted by pericytes and astrocyte endfeet, and their respective basement membranes, as watchmen (Figure 1). In 2014, our knowledge of the BBB drawbridge has turned 100 years. Although some of the experiments that led to the discovery of the BBB may be known, it is certainly less well known that this pillar of knowledge in neuroscience is intertwined with the history of a deadly tropical disease, sleeping sickness or human African trypanosomiasis. This disease is caused by the extracellular protozoan African trypanosome, which is capable of entering the CNS by crossing the BBB.

We owe the discovery of the BBB to attempts to deliver drugs to the brain for treatment of African trypanosome infections. These studies also contributed to the later development of the concept of endocytosis, which is a core principle in cell biology and in neurobiology. Our understanding of regulated endocytosis in the BBB and in trypanosomes is now rapidly evolving in parallel, and it is largely forgotten that such findings originated from the same studies 100 years ago. In this review, we highlight novel findings on how parasites can enter the CNS and persist in the CNS parenchyma, focusing on the BBB in view of current studies on trypanosome–brain interactions. Because trypanosomes were pivotal to the discovery of the BBB, we highlight how BBB research may repay trypanosome research. This also opens questions on cell trafficking mechanisms in and out of the BBB, on the importance of endocytotic mechanisms for drug targeting, and on the potential role of disturbed BBB gating in the pathogenesis of neurodegeneration.

## The story of rejected bullets

Central to development of the ‘chemotherapy’ concept at the beginning of the 20th century was the use of colored drugs to visualize and distinguish microbes (to kill) from host cells (to spare). African trypanosomes represented the first target for chemotherapy, and drugs called ‘trypan dyes’ (a name derived from the parasites) were used. These dyes not only played a crucial role in the detection of the BBB, but also became important markers of cell viability because they do not pass intact cell membranes, playing a key role also in the concept of endocytosis.

### Trypanosomes and African trypanosomiasis: the search to cure a lethal infection

Between 1901 and 1903, an outbreak of the lethal disease sleeping sickness affected about 80% of the population on the Northern side of Lake Victoria. A commission (the epidemiologist Cuthbert Christy, the parasitologist George Low, and the bacteriologist Aldo Castellani) was sent to Entebbe, Uganda, in July 1902. Using a hand-driven centrifuge and a moderately powerful microscope, Castellani observed trypanosomes in the cerebrospinal fluid (CSF) of patients [1]. This was surprising because trypanosomes were at that time only known to be the cause of ‘nagana’, a disease endemic among south-African cattle, as discovered by Sir David Bruce. The causative agents were called after him, *Trypanosoma* (*T.*) *brucei*, a denomination derived from the Greek ‘trypanon’ (drill), probably due to the rotating movement of the parasite, which is equipped with a flagellum, in the bloodstream.

Because this was the time of colonialism, it became of public interest to find a remedy. Considering that protozoa could be more susceptible to chemical agents than bacteria, Paul Ehrlich (1854–1915) engaged in research based on the concept of chemotherapy he had introduced [2]. Inspired by treatment of malaria with quinine and by the attempts, by Laveran and Mesnil, to treat with an arsenic compound mice infected with trypanosomes from cattle suffering from ‘nagana’, Ehrlich used trypanosomes as his first target [2]. A donation by Mrs Franziska Speyer had provided the funding for the chemotherapy institute Georg Speyer-Hause in Frankfurt devoted to Ehrlich's research to replace empirical therapy with a ‘chemotherapia specifica’ by which parasites could be killed without causing major damage to the organism [3]; thereby, ‘money’ could be added to ‘patience’, ‘skill’, and ‘luck’, the four most important factors for fruitful research as defined by Ehrlich [4].

### To see or not to see: trypan dyes

To enable distinction between targets (microbes) and non-targets (host cells), Ehrlich searched for suitable colored compounds that could stain and kill parasites like ‘bewitched bullets’. Methylene blue, which was in common use, was found to be too toxic to the host. In his first series of experiments, Ehrlich therefore turned to the so-called azo dyes derived from benzopurpurins. After testing several azo dyes, a water-soluble red compound was found to be trypanocidal with a tolerable low toxicity and was christened ‘trypanroth’ (‘trypan red’) [2]. For his studies, Ehrlich received as gifts guinea pigs infected with a horse pathogenic trypanosome strain, *T. equinum*, which causes ‘mal de caderas’ in South America. Mice injected with blood from these guinea pigs died within 4–5 days, but injection of trypan red within 3 days of infection could cure the mice [2]. This marked the first successful ‘chemotherapy’: killing a microbe with a specifically designed and selected drug.

However, relapses of the infection occurred up to several months after treatment, and attempts to treat *T. brucei* strains failed, as did trials with about 50 alternatives to trypan red. The studies therefore came to an end in 1905 [3]. Ehrlich then focused, until his death, on the treatment of syphilis with the arsenical compound Salversan. In the meantime, blue and violet trypan dyes had been synthesized by the Bayer Company. These dyes showed potentially more promising effects, as observed by Nicolle and Mesnil at the Pasteur Institute [3,5]. Among them, trypan blue became a vital staining agent destined to be a protagonist in the discovery of the BBB.

### CNS regions ‘white as snow’ turning blue: the discovery of the BBB

**Figure 2:**
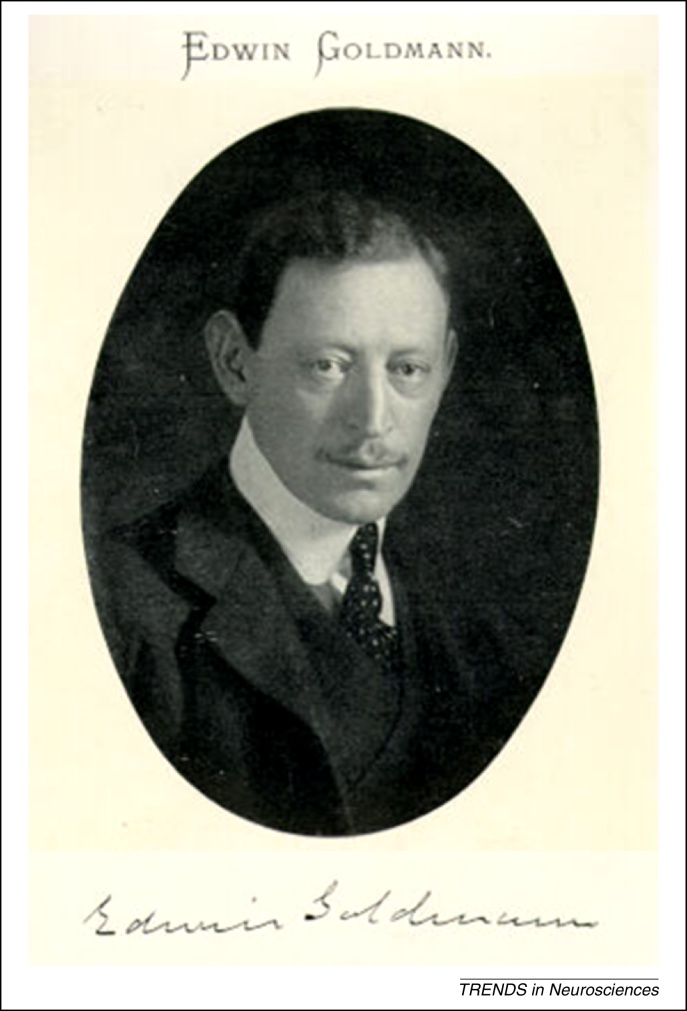
Portrait of Edwin Ellen Goldmann. Born in 1862 in Burgherdorp, South Africa, Goldmann died of cancer in 1913 in Freiburg im Bresgau, Germany, 2 months after his contribution to the discovery of the blood–brain barrier (BBB) [7]. Goldmann is not described as a student or associate of Ehrlich, but they should have been in contact. Goldmann's experiments that led to the detection of the BBB were done with the financial support of the late Sir Julius Wernher and others, and with the technical and intellectual assistance of Marie Schmelzer, a lay worker. (Image in the public domain: http://de.wikipedia.org/wiki/Edwin_Goldmann).

Edwin E. Goldmann (1862–1913) (Figure 2), a native of South Africa, studied medicine in London followed by visits to German universities (Breslau and Frankfurt) before devoting himself at a lay worker's institution in Freiburg as a surgeon of tumors. In his studies on experimental tumors, Goldmann performed vital staining using dyes devised by Ehrlich. Based on this work, he noted that trypan blue showed the lowest toxicity and spread fastest in the body. In his first series of experiments, Goldmann observed that following systemic injections of trypan blue in several species (frogs, mice, rats, guinea pigs, rabbits, dogs, and monkeys) the whole animals were rapidly stained blue. However, the brain and spinal cord remained ‘white as snow’, with the exception of the choroid plexus [6]. The question therefore arose: was the lack of staining of the CNS due to lack of dye affinity or to a failure of dye entry into the brain parenchyma? This question was solved in the next set of experiments on young rabbits [7].

**Figure 3:**
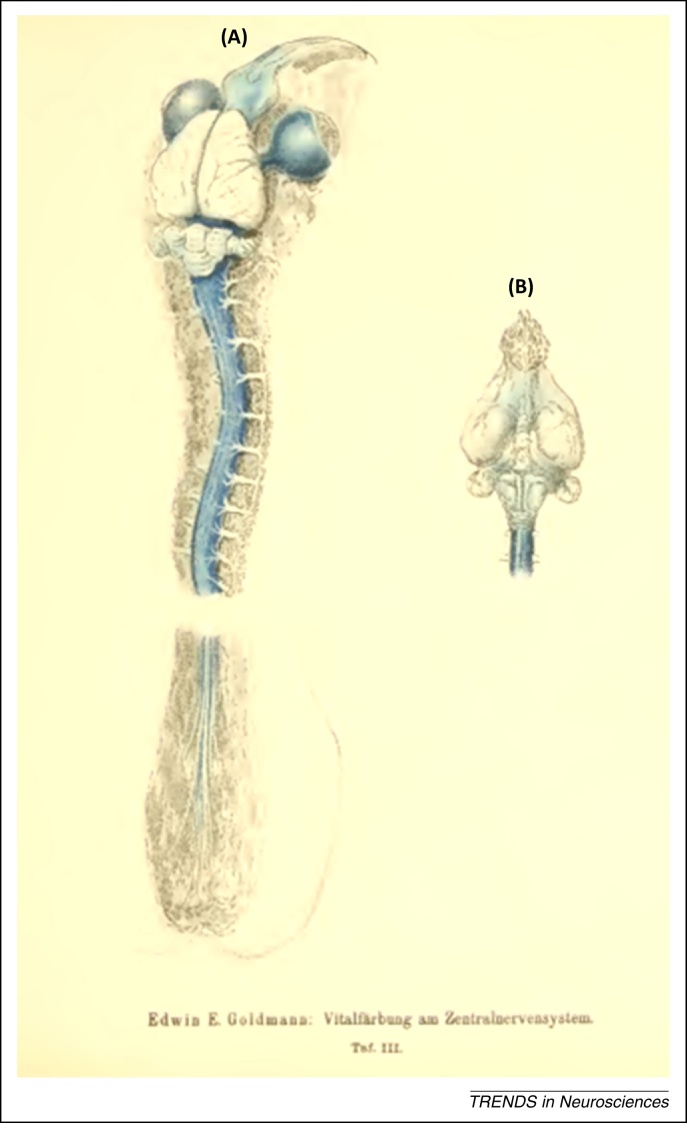
Illustration of Goldmann's seminal discovery of the blood–brain barrier (BBB) in 1913. Overview of the central nervous system (CNS) of a young rabbit injected with 0.5 ml of a 0.5% trypan blue solution into the lumbar ‘cul-de-sac’. Table III from [7]; **(A)** dorsal and **(B)** ventral view*.* The whole spinal cord and brainstem are stained blue, as are the optic nerves, sclerae, and olfactory areas (i.e., the regions to which the dye could rapidly flow). Note that Goldmann did not perform injections of the dye into the cerebral ventricles or the brain parenchyma. The illustration clearly shows that the lack of trypan blue staining of the CNS following systemic injections of the dye was not due to lack of dye affinity for the CNS, but instead to lack of dye penetration into the brain parenchyma from the blood and choroid plexus, and therefore to a permeability problem, that is, to the existence of a blood–brain and blood–cerebrospinal fluid (CSF) barrier. By two-photon laser scanning microscopy, it has been recently visualized that substances of low molecular weight (such as trypan blue, which in the CSF is not bound to any serum protein), which circulate in the CSF, rapidly enter the CNS interstitium from both the pial surface and spaces around arteries/arterioles, whereas those of high molecular weight are confined to the paravascular spaces [41].

Following injections of trypan blue into the subarachnoid space at lumbar level or in the cisterna magna, the CNS regions to which the dye could rapidly flow (spinal cord, brainstem, cerebellum, and basal forebrain) turned out to be stained blue (Figure 3). This showed that lack of CNS tissue staining after systemic injections of the dye was due to lack of dye penetration from the blood into the CNS and not to lack of dye affinity. The existence of a blood–brain and blood–CSF barrier was for the first time clearly visualized and demonstrated [7].

### Enemies hiding behind the BBB

Why could Ehrlich not prevent relapses by treating trypanosome-infected rodents with trypanocidal trypan dyes? The answer to this question came, after decades, from a series of experiments by Frank Jennings and collaborators in Glasgow [8]. They treated *T. brucei-*infected mice with the trypanocidal drug diminazene aceturate (an aromatic diamidine used in veterinary medicine to treat early-stage African trypanosomiasis). Although the parasites were eliminated from the blood, the infections relapsed in all mice 4–7 weeks later. Injections of homogenates from the brain of treated mice, taken before the relapses, could successfully transmit the infection to naïve mice, whereas injections of other tissues or blood could not. This meant that the parasites could hide in the brain behind an intact BBB, being inaccessible to diminazene, which does not cross the BBB. The findings imply that Ehrlich's failure in preventing relapse was due to the fact that trypan dyes do not cross the BBB.

This observation raises three questions. (i) How can trypanosomes enter the brain parenchyma and persist behind an intact BBB? (ii) How can trypanosome growth in the brain be controlled so that the parasites stay alive and do not cause a rapidly overwhelming infection? (iii) How can the unicellular trypanosomes exit the brain to cause a relapse?

## Challenges in parasite trafficking in and out the brain: fooling the drawbridge?

### Parasite entry and brain immune response

In experimental rodent models, *T. brucei* subspecies appear in the choroid plexus soon after infection. At the same time, parasites lodge in the circumventricular organs (CVOs) and dorsal nerve root ganglia which, like the choroid plexus, have fenestrated endothelia and lack tight junctions. Trypanosomes also appear in the leptomeninges early after infection [9]. However, it is not clear whether lodging in the meninges reflects passage from the choroid plexus and subsequent spread in the CSF, as suggested by Goldmann [7] and recently by Wolburg *et al.* [10], or direct crossing of leptomeningeal vessels, more permeable to macromolecules than brain parenchymal vessels [11]. The lack of an astrocyte-derived parenchymal basement membrane in meningeal vessels may also facilitate the passage of pathogens into the meninges and subarachnoid space.

**Figure 4:**
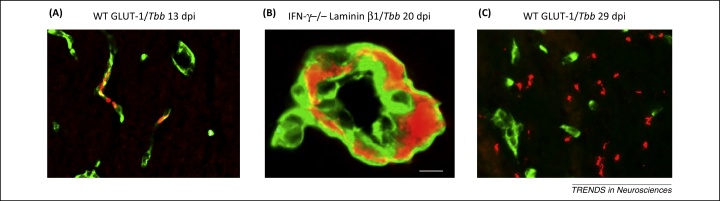
African trypanosomes in the brain. **(A)***Trypanosoma brucei brucei* (*Tbb*; red) within brain capillaries (green; cerebral endothelial cells are stained by glutamate transporter-1, GLUT-1) in wild type (WT) mice early after infection (13 days post-infection, dpi). **(B)** Accumulation of trypanosomes (red) and white blood cells (seen as silhouettes) as ‘cuffs’ within basement membranes (green; labelled with antibodies against laminin β1 that is present in both the endothelial and astrocytic basement membranes) of a larger intracerebral vessel in an interferon-γ (*IFN-γ*)-deficient mouse at 20 dpi. **(C)** Numerous extravascular trypanosomes (red) in the white matter at the late stage of infection (green; cerebral endothelial cells). The plate illustrates that passage of trypanosomes from the blood into the brain through the outer parenchymal basement membrane is dependent on immune response molecules induced by the infections. Scale bar: 10 μm. Reproduced, with permission, from [76].

Further *T. brucei* passage across the cerebral microvasculature (Figure 4) shows large temporal variations dependent on the host species and strain, as well as on the *T. brucei* subspecies. Remarkably, brain invasion by trypanosomes is not related to the level of parasitemia but to the host immune response [12]. For instance, treatment with minocycline, a drug that reduces the immune response in the brain, also, paradoxically, reduces parasite neuroinvasion [13]. In particular, the T helper 1 (Th1) immune response increases trypanosome neuroinvasion: in the absence of interferon (IFN)-γ and T cells, parasite entry into the brain parenchyma is greatly reduced although parasitemia levels may be increased [9].

It should be emphasized that invasion of T cells and trypanosomes occurs at the postcapillary venules [9], where there is a perivascular space, and not at the capillary level, which is usually the focus in studies on BBB properties [14]. To invade the brain parenchyma, circulating T cells have to attach to endothelial adhesion molecules, and then pass either through openings of tight junctions or transendothelially to meet the next hurdle of the basement membranes [15,16]. Whether trypanosomes pass *in vivo* via tight junction openings or transcytosis remains elusive; both possibilities have been suggested *in vitro* [17,18]. *In vitro* models of the BBB, however, do not reflect the complexities of the *in vivo* situation [19], not the least those of postcapillary venules.

The endothelial cells of cerebral postcapillary venules are enwrapped by two basement membranes: the endothelial membrane and the parenchymal basement membrane, also called the astrocytic basement membrane [14,16]. T cell penetration depends on the composition of laminin molecules in these membranes. Laminin α5 chains in the basement membranes around capillaries do not permit T cell invasion, whereas laminin α4 chains, at postcapillary sites, do. In addition, the astrocytic basement membranes (laminin α1 and α2 chains) must be focally ‘opened’ by activated matrix metalloproteases to allow T cell passage; if this does not occur, T cells wait ‘on hold’ as cuffs around the vessels [14,16].

Trypanosomes closely follow this series of events in their brain invasion [9], which is initiated by molecules released by the innate immune response. Via an initial release of low levels of C–X–C motif chemokine 10 (CXCL10), the innate immune response communicates with the adaptive immune response [20]. This chemokine may facilitate the recruitment of trypanosome-sensitized T cells into the brain to recognize trypanosome antigen-presenting cells, for example, dendritic cells. Sensitized T cells are thereby activated to secrete IFN-γ [20], which stimulates CXCL10 secretion accelerating the process of trypanosome neuroinvasion [21].

This chain of events is similar to that described for brain infections with lymphocytic choriomeningitis virus [22], pointing to a more general role of CXCL10 in the crosstalk between the innate and adaptive immune response in the CNS. In addition, CXCL10 is not only needed for the recruitment and/or retention of antigen-specific T cells in the brain, but can also enhance T cell migration speed, thus shortening the time to find rare targets. This is the so-called ‘Lévy walk’, observed during brain infections with the intracellular parasite *Toxoplasma gondii* [23]. How the extracellular trypanosomes move within the brain parenchyma in relation to T cells and dendritic cells is, therefore, intriguing to determine.

An accelerated brain invasion of trypanosomes would not be compatible with survival of the host, needed for transmission to other individuals through the parasite vector (the tsetse fly). Therefore the parasite passage across the BBB must be controlled. However, there is a gap in knowledge on molecular closure mechanisms of the CNS gate after its opening. Interestingly, administration of exogenous nitric oxide can reduce vascular leak in cerebral malaria [24–26], and may, speculatively, also play a role in ‘closing the door’ behind trypanosomes entering the brain parenchyma.

### Surviving within the CNS castle: parasite persistence

How do trypanosomes persist in the brain? Although the BBB protects them from antibodies circulating in the bloodstream, the CNS castle may not be secure for the parasites, whose survival may be endangered by still undefined trypanotoxic constituents circulating in the CSF [10]. The search for such host-derived factors in the CSF could lead to the identification of molecules to combat the parasite. In addition, the CNS castle is equipped with devices against enemies and, in particular, a number of neuropeptides are antimicrobial, showing high affinity to bacterial, but not mammalian, cell membranes [27]. For example, neuropeptide Y produced in the olfactory epithelium [28] can prevent bacteria from entering the CNS along olfactory pathways [29]. *In vitro*, several neuropeptides (e.g., vasoactive intestinal peptide, adrenomedullin, and urocortin) are trypanolytic [30], but only at relatively high concentrations (μM), and their role *in vivo* remains to be investigated. The neuropeptide substance P can, however, at physiological concentrations reduce the severity of trypanosome-induced neuroinflammation [31].

To survive for extended periods of time in the brain castle without demolishing it by killing the host, the life of trypanosomes may be balanced by both growth-inhibiting and -promoting molecules in the environment. During their persistence in the brain, trypanosomes have been observed mainly in the olfactory bulbs and cerebellum [32,33], where turnover of small granular neurons occur. Cell growth-promoting factors in these regions could potentially also favor pathogen survival, an interesting topic that needs to be investigated. In support of this assumption, it should also be considered that, at the periphery, trypanosomes can persist in the testes [34], which are equipped with a blood–testis barrier and produce a number of cell growth-regulating factors [35].

### How can cells exit the brain? Escape routes

**Figure 5:**
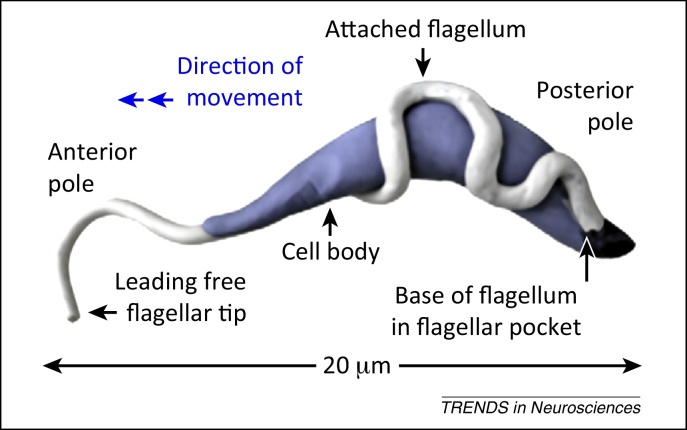
Swim or sink: trypanosome as a drill. Illustration of *Trypanosoma brucei* cell architecture. The *T. brucei* human pathogenic subspecies are spread by the vector (tsetse fly) and exist in the mammalian host in two forms, the rapidly dividing (5–6 hours) slender form and the nondividing stumpy form, which can be transmitted back to the fly. Slender trypanosomes secrete a molecule that induces the transformation into the stumpy form [77] to prevent overwhelming infections that could rapidly kill the host. Trypanosomes prosper in the bloodstream. Slender trypanosomes are pulled forward in rotational movements, like drills, by the planar beat of their flagellum and reach their maximum velocity in the cell density of streaming blood [78]*.* If cell density increases, the flagellar beats are reversed and trypanosomes swim backward not to get trapped [78]. The motility of trypanosomes in the dense brain tissue remains to be explored. Reproduced, with permission, from [78].

A proportion of trypanosomes should remain in proliferating (slender) form to be able to expand and cause relapses upon exiting the brain (Figure 5). *T. brucei brucei* can cross the BBB from the abluminal to the luminal side of endothelial cells *in vitro* [36], but this remains to be verified *in vivo*. The direction of parasite swimming, due to movement of the flagellum forward or backward according to tissue densities (Figure 5), could also influence trypanosome trafficking in and out the brain.

Although T cells facilitate trypanosomes entry into the brain, different mechanisms may be involved in trypanosome exit from the brain. In general, T cells are killed in the tissues, for example, by Fas ligand-induced apoptosis, when their mission is accomplished [37]. Interestingly, in rabies, a severe viral CNS infection, the killing of T cells is turned on prematurely by the rabies virus in neurons to evade virus elimination [38]. Antigen-presenting cells may cross the BBB after injection into the brain parenchyma [39], and a population of such cells can protrude dendritic extensions across the BBB into the vascular lumen [40]. It is not known, however, whether such a mechanism could provide an escape route for trypanosomes into the blood.

Goldmann [7] noted that trypan blue injected into the CSF of dogs diffused into the brain along perivascular spaces, but also appeared in deep cervical lymph nodes, suggesting a direct communication between brain and lymphatics. Therefore, a lymphatic drainage of the brain was proposed in addition to the classical drainage of CSF into the blood through arachnoid villi and granulations. The CSF may be reached by molecules from the brain parenchyma via paravenous drainage pathways. Although CSF influx into the interstitial spaces of the brain parenchyma is partly driven by arterial pulse waves along periarterial spaces, outflux of soluble molecules is driven by the water channel aquaporin-4 in astrocytic endfeet [41]. Cortical interstitial spaces increase during sleep facilitating the clearance of soluble metabolites [42], but it is not known whether interstitial spaces could become sufficiently large to facilitate the propelling of cells, such as trypanosomes, into the CSF.

Once in the CSF, antigen-presenting cells could reach the nasal and lumbar lymphatics via the channels that drain CSF from the subarachnoid space along olfactory and spinal nerves [43]. This could provide an exit route for trypanosomes, especially from the olfactory bulbs, where they may persist before relapses [32,33] having access to nasal lymphatics through the cribriform plate.

An alternative pathway suggested for soluble molecules would lead directly to cervical lymph nodes from the brain parenchyma along basement membranes between smooth muscle cells of arterioles and arteries by contrary waves following arterial pulse waves [43]. This, however, is an unlikely pathway for the escape from the brain of cells, including trypanosomes, which have not been seen along such basement membranes.

### The host–pathogen duel manipulates brain function

What do trypanosomes do in the brain? A prominent sign of sleeping sickness is represented by sleep/wake disturbances, with periods of sleep during daytime and insomnia at night, as well as a fragmented sleep pattern with narcolepsy-like sleep episodes [44,45]. These alterations differ from the somnolence that occurs in so-called ‘sickness behavior’ during systemic infections.

Trypanosomes produce prostaglandin D2, which can induce slow-wave sleep following intracerebroventricular injections, and the release of this molecule could play a role in sleep changes during the infection [10]. However, the total sleep time during a 24-hour period is not increased in *T. brucei*-infected humans and rodents [44,45]. The unique, early localization of trypanosomes to CVOs followed by invasion of parasites and inflammatory cells into diencephalic areas could affect the functioning of the master circadian clock (the suprachiasmatic nucleus) and sleep/wake-regulating cell groups in the hypothalamus [9]. Whether sleep/wake alterations or other behavioral changes caused by *T. brucei* infections pose any advantage for parasite spread by the tsetse fly remains to be clarified [10]. This question is of interest to the current debate as to whether behavioral changes caused by persistent parasitic brain infections can favor parasite survival, for example, in *Toxoplasma gondii* infections [46]. This parasite may manipulate intermediate host behavior to enhance the risk of the host to become victim of a predator and, thus, increase its own dissemination [47,48].

## The key role of endocytosis

### Endocytosis in trypanosomes

As mentioned above, trypan blue became a classical marker for cell viability because it cannot pass intact cell membranes and does not stain most living cells. The question how trypan dyes enter trypanosomes and kill them has recently found an explanation. Trypanosomes show a massive endocytosis, which enables them to remove antibodies against the variant surface glycoproteins (VSG) that cover their surface. Endocytosis occurs in large clathrin-coated vesicles in the so-called flagellar pocket at the posterior end of the parasite [49] (Figure 5). The whole pool of VSG can be turned over within minutes and the speed is accelerated by the parasite swimming in the bloodstream [50]. The removal of attached antibodies against VSG by endocytosis could be a means to evade the humoral immune response, in addition to the pre-programmed switches of the VSG coats [51]. This makes conventional VSG-specific antibodies, which depend on complement activation, inefficient in killing trypanosomes. Because trypan blue is incorporated by endocytosis, the dye is taken up by trypanosomes in large amounts and labels them rapidly blue, in contrast to most host cells with a much lower level of endocytosis. Endocytosis at the parasite flagellar pocket is currently a target for the development of antitrypanosome drugs [52] (Box 1).

### Endocytosis and pericytes in the BBB

Tight junctions linking cerebral microvascular endothelia and the scarcity of fluid-phase endocytosis are defining features of the BBB maintenance of brain homeostasis [53,54]. Thus, in contrast to the exceptionally high level of endocytosis in bloodstream trypanosomes, which prevents antibodies from attacking them, cerebral endothelial cells are endowed with remarkably low endocytosis or transcytosis, which also prevents circulating antibodies from entering the brain.

Pericytes, which are mesenchymal-derived cells, are key regulators of transcytosis in cerebral endothelia [55]. Pericytes encircle cerebral endothelial cells, enwrapped in the same basement membrane (Figure 1), and can affect transcytosis in these cells during development [56], in addition to their role in the structural integrity and vasodynamic capacity of the BBB [57,58]. Transcytosis is highly enhanced, while tight junctions are preserved, in pericyte-deficient mice, indicating that pericytes regulate the level of transcytosis also in adult mice [55,59] (Figure 1).

In addition to the control of transcytosis, pericytes of the BBB can contribute to the ‘opening’ of cerebral endothelial tight junctions (Figure 1) through the activation of a proinflammatory cyclophilin A–matrix metalloprotease-9 pathway [60]. Interestingly, pericytes can be under the attack of viruses, such as HIV and Japanese encephalitis virus, which cause infections associated with neurodegeneration [61,62]. The question therefore arises: can pericyte-regulated processes in the BBB contribute to dysfunctions or destructions of neurons?

## Alterations of the BBB as a gateway to neurodegeneration?

Changes in BBB permeability, which could reflect functional alterations of pericytes and tight junction opening, have been recently suggested to precede neurodegeneration and cognitive decline during aging [59], as well as in apolipoprotein E (*APOE*) gene-manipulated mice [60]. Putative mechanisms for these events include passage, across openings in tight junctions, of serum proteins that may be toxic to neurons, diminished brain capillary perfusion leading to hypoxia, and loss of pericyte-derived trophic molecules that promote the survival of cerebral endothelial cells [59,63–66].

In contrast to these observations, extensive clinical search for signs of increased BBB permeability by CSF/serum protein as well as histological and neuroimaging studies in patients affected by Alzheimer's disease (AD) have failed to provide clear-cut evidence for BBB disruption in this major neurodegenerative disease [67]. However, several findings point to a dysfunction at the level of transcytosis across the BBB of amyloid-β (Aβ) molecules, which are thought to play a pathogenetic role in AD. Such dysfunction could lead to accumulation of misfolded Aβ around intracerebral vessels, reflecting either a deficit in Aβ clearance from the brain across the BBB or an increased influx from the blood. Lipoprotein receptor-related protein 1 (LRP-1) [68] and the multidrug transporter P protein (Pgp) [69,70] can both promote Aβ efflux into the bloodstream, whereas the receptor for advanced glycation endproducts (RAGE) can transport soluble Aβ from the blood into the brain [71]. A disturbed balance between these influx and efflux processes could lead to pathological accumulation of toxic Aβ peptides in the brain and has therefore been implicated in AD pathogenesis [67,72].

However, Aβ may also be cleared from the brain through other mechanisms. For instance, the soluble Aβ form can be cleared experimentally from the brain through paravenous spaces to the CSF rather than across the BBB [41]. Aβ clearance could also occur along the above-mentioned cerebral arterial basement membranes [43].

Finally, concerning current challenges for mechanisms of neurodegeneration, studies on trypanosome brain infections may be relevant for the extensive and longstanding debate on the contribution of persistent or chronic inflammation in the CNS to the pathogenesis of neurodegenerative diseases [73,74]. Chronic trypanosome infections could be of interest for studies on putative factors that may tilt the balance towards neurotoxicity or neuroprotection. For example, aspirin (sodium salicylate) treatment exacerbates brain neurodegeneration in trypanosome-infected rats [75], demonstrating that chronic neuroinflammation can be neuroprotective.

## Concluding remarks

Inflammatory cells and molecules trafficking to, within, and out of the brain, as discovered by the use of African trypanosomes and trypan dyes 100 years ago, have now reached the frontline of research on brain disorders, raising many questions (Box 2). Neuroinflammation, a term introduced only 20 years ago, is thereby currently on stage due to its importance in healthy brain aging and in neurodegenerative diseases, whereas the field of brain infections is relatively neglected in neuroscience, considered mainly a domain of microbiology. However, the fight between a competent BBB and pathogens searching for secure niches in the brain microenvironment, where they can escape the bullets of the immune system, has yielded key insight into CNS health and disease. Interestingly, these studies can contribute to unravelling mechanisms by which T cells can pave the way to BBB crossing of other elements, as well as mechanisms by which cells could exit the brain, and those by which inflammation could be switched off.

Last but not least, targeted brain delivery of drugs (Ehrlich's ‘bewitched bullets’) is still an open field of active investigation, using, for example, nanobodies or nanoparticles as minute carriages to fool the BBB drawbridge (Box 1). However, despite this progress, safe and effective therapies for brain involvement in African trypanosomiasis remain to be found after more than a century.

## Box 1 Trypanosome neuroinvasion governs therapy decisions to kill the parasite or the host

*Decisions on life or death*

The crossing of an intact BBB by African trypanosomes is an important consideration for therapy decisions. Presently, finding trypanosomes and/or more than five white blood cells in the CSF is used as marker of parasite neuroinvasion. Suramin and pentamidine are drugs of choice for treatment of early-stage *T. brucei rhodensiense* and *T. brucei gambiense* infections, respectively, but they do not cross the BBB. More toxic drugs such as arsenical compounds (which can be lethal), eflornithine, or eflornithine in combination with nitrofurmox are in use for late-stage therapy [79,80]. However, experimental findings have shown that trypanosomes can appear in the meninges (and the CSF) early after infection [10,32,81], when the animals are still sensitive to early-stage drugs.

Signs of nervous system involvement may be misleading for the staging of sleeping sickness, because some of them (neuropathic pain and sleep changes) may appear early after infection [82] when in experimental models trypanosomes are still mainly localized to dorsal root ganglia and CVOs and accessible to drugs that do not cross the BBB. Better objective clinical measures, including biomarkers for disease staging, are therefore a priority. Neopterin, which is a stable product of IFN-γ-stimulated macrophages [83,84], and the IFN-γ-inducible chemokine CXCL10 could be biologically meaningful biomarkers [85–87], because IFN-γ facilitates trypanosome passage across the BBB [9].

*Targeting trypanosome endocytosis*

Endocytosis at the trypanosome flagellar pocket is currently being explored for the targeted delivery of trypanocidal molecules [52]. The receptor for suramin (the colorless derivative of trypan dyes synthesized in 1916, kept as a war secret and released for clinical use only in the early 1920s) has been identified in an invariant surface glycoprotein at the flagellar pocket. Following endocytosis, this glycoprotein is delivered to lysosomes where suramin, once free, inhibits cellular processes, similarly to trypan blue. This explains why trypan compounds affect trypanosomes (high endocytosis) but not host cells (low endocytosis), that is, the basis for Ehrlich's chemotherapy concept [52]. New drugs are now developed, and these include nanobodies that elicit trypanocidal effects by endocytosis inhibition [88–90], acting as the ‘bewitched bullets’ Ehrlich envisaged.

## Box 2 Outstanding questions

• How can invading extracellular pathogens, such as trypanosomes, hijack the precisely regulated multi-step infiltration of white blood cells across the BBB, and by which mechanisms can the BBB gateway be closed behind entering cells?
• How is trafficking of pathogens and inflammatory cells within the brain parenchyma regulated while they criss-cross the sensitive nervous tissue?
• How are the immune responses in the brain switched on and off to balance effects on invading microbes and the nervous tissue? Which factors can tilt the balance to elicit relapse of infections, recovery, or irreversible post-inflammatory reactions?
• Which inflammatory mediators in cerebral endothelial cells, pericytes, and macrophages/microglia contribute to neurodegeneration or protect against it? Can safe anti-inflammatory therapies of brain infectious diseases be designed to kill the pathogen while promoting the survival of neurons?
• Do astrocytic endfeet at the BBB bridge interactions between synaptic activities and cerebral endothelial immune reactions, i.e., do synaptic activity and immune responses interact for reciprocal regulation during infection/inflammation?
• Can transcytosis in cerebral endothelial cells be manipulated to promote selective delivery of drugs across the BBB? Can transporter ligands that efficiently detach after BBB crossing [91] be developed? Can pericytes be targets for therapies that aim at inhibiting microbe invasion and promote targeted drug delivery to the brain?
